# Metastasectomy for extracalvarial renal cell carcinoma

**DOI:** 10.1002/ccr3.8967

**Published:** 2024-06-05

**Authors:** Rahim Abo Kasem, Karan Joseph, Adnan Shaik, Angela Downes, M. Burhan Janjua

**Affiliations:** ^1^ Department of Neurosurgery Medical University of South Carolina Charleston South Carolina USA; ^2^ Department of Neurosurgery Washington University School of Medicine St. Louis Missouri USA; ^3^ School of Medicine University of Missouri‐Kansas City Kansas City Missouri USA; ^4^ Department of Neurosurgery University School of Colorado Denver Colorado USA

**Keywords:** extracalvarial metastasis, metastatic renal cell carcinoma, preoperative embolization, surgical resection, titanium cranioplasty

## Abstract

**Key Clinical Message:**

Palliative surgical resection of extra‐calvarial metastatic lesions from renal cell tumors is crucial for controlling metastatic spread, improving quality of life, and preventing associated morbidity. Careful surgical planning, including selective preoperative embolization and controlled resection around critical structures such as the sagittal sinus, is essential for successful outcomes. Cranioplasty with Titanium mesh and bone cement post‐resection can provide symptomatic relief, better cosmesis, and overall improved quality of life.

**Abstract:**

Renal cell carcinomas are aggressive tumors with distant systemic disease. The calvarium appears to be an unusual and rare site for distant metastasis. The treatment modalities are challenging and out of the normal realm for the management of these tumors. We report a case of a 63‐year‐old woman with a previous history of nephrectomy who presented with symptoms of severe headaches, and swelling of bi‐frontal and bi‐parietal scalp regions due to multifocal extracalvarial disease. Preoperative bilateral superficial temporal artery embolization was performed to control the intraoperative bleeding. Surgical technique has been described with the critical steps involved, and a literature review has been conducted. Palliative tumor resection surgery was performed to improve the patient's quality of life as well as to confirm the histopathological diagnosis. Gross total resection of the extracalvarial metastatic tumor was achieved. Biopsy confirmed renal cell tumor with the clear cell subtype. The patient recovered well from her surgery with slow healing of the scalp wound. At 6‐month follow‐up, no recurrence of the extracalvarial disease was observed on serial imaging. Extracalvarial metastasis is a rare presentation in renal cell carcinoma. Considering the inherent radioresistant nature of the tumor, palliative surgical resection can be offered to control the metastatic spread, relieve agonizing pain symptoms, and to improve the quality of life. Preoperative embolization helps to decrease intraoperative blood loss. Moreover, palliative surgical resection of extracalvarial diseases helps to treat the metastasis as well as avoiding the associated morbidity that may occur if left untreated.

## 1 INTRODUCTION

Renal cell carcinoma (RCC) is a common malignancy with the highest incidence found in developed countries and a predilection for men (0.7 per 100,000 people worldwide for men).^1^ It arises from the renal tubular epithelial cells by various mechanisms.^2^ Risk factors include genetics and lifestyle factors such as smoking, obesity, and hypertension.^1^, ^2^, ^3^ Major subtypes have been described, including clear cell renal cell carcinoma (CCRCC), papillary renal cell carcinoma (PRCC), and chromophobe renal cell carcinoma (CRCC). CCRCC seems to be the most common, comprising 65%–70% of total renal cell carcinomas.^2^, ^4^ Inactivation of the von Hippel–Lindau tumor suppressor gene on the short arm of chromosome 3 is particularly involved in the pathogenesis of CCRCC in both sporadic and inherited disease presentation.^2^, ^4^, ^5^ About 30% of patients diagnosed with RCC already have metastatic disease.^1^, ^2^ It is believed that about one‐third of all patients with RCC will eventually develop metastatic disease.^1^ Metastasis from RCC can exhibit a variety of clinical presentations, Common sites of metastasis include the lungs, liver, bones, and less frequently, the brain and other distant organs. Pulmonary metastases often present with respiratory symptoms and appear as nodules on imaging,^6^ Osseous metastases frequently lead to skeletal complications such as bone pain and pathologic fractures.^7^ while gastrointestinal metastasis may manifest with abdominal discomfort and lesions on imaging.^8^, ^9^ Additionally, RCC may involve the adrenal glands, contralateral kidney, or venous system, necessitating comprehensive staging and surveillance.^1^, ^2^ Renal cell carcinoma metastases usually spread by lymphatic or hematogenous routes with the venous system acting as a pathway to reach systemic circulation.^2^ However, the calvarium appears to be an unusual site and is a rare metastatic presentation.^2^, ^3^, ^4^

Metastatic tumors are the most common cause of calvarial lesions in adults. Patients with calvarial metastatic tumors are usually asymptomatic until the lesions become extracalvarial and may present with headaches, scalp tenderness, lethargy, scalp ulceration, or neurological deficits (if invasive to the cerebral cortex).^1^, ^10^, ^11^, ^12^, ^13^, ^14^, ^15^

Similarly, our case is unique in its presentation. Treatment modalities are quite challenging and out of the normal realm for management of neoplasms of the calvarium. Authors discuss the role of palliative surgery in these patients both with and without systemic disease control.

This report outlines our surgical approach in managing a 63‐year‐old female patient with renal cell calvarial metastasis, aiming for palliative relief from extracalvarial disease symptoms while obtaining histopathological tissue for tailored immunotherapy guidance.

## 2 CASE REPORT

### 2.1 Patient history and clinical presentation

In this case report, a female patient aged 63, previously diagnosed with metastatic renal cell carcinoma (clear cell type) and a history of nephrectomy, who is currently undergoing intermittent renal dialysis, manifested incapacitating headaches, scalp tightness, tenderness, and bilateral swelling in the bi‐frontal and bi‐parietal scalp regions. The general physical examination yielded unremarkable findings, while we observed a palpable firm mass measuring 3 × 3 cm over the bregma (anterior scalp) and in biparietal scalp regions, with the right side exhibiting a greater prominence, which was found on thorough neurological examination.

### 2.2 Diagnostic imaging and assessment

**FIGURE 1:**
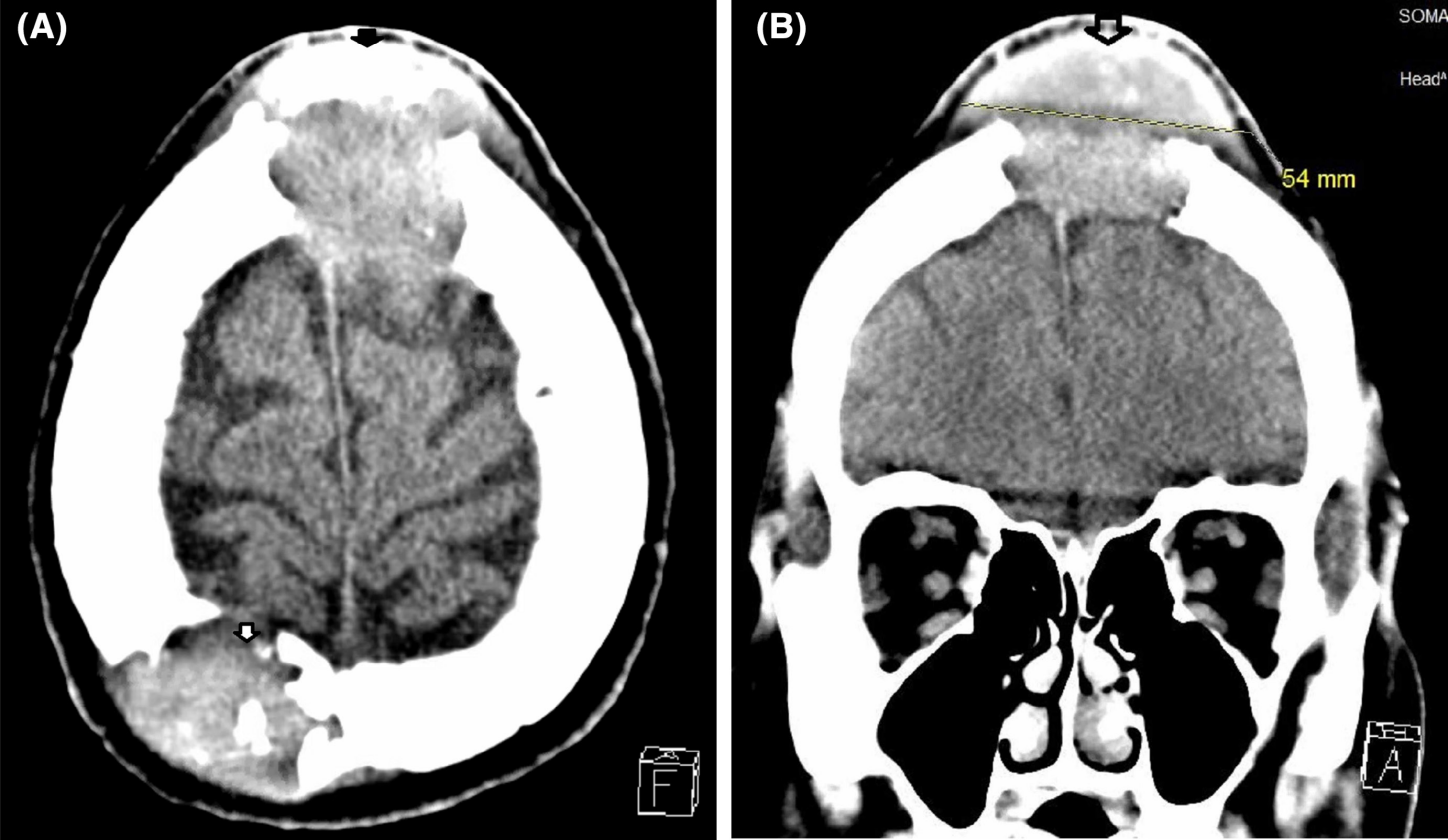
(A) Preoperative head CT scan axial view depicting a right sided parietal extracalvarial metastatic lesion. (B) Preoperative head CT scan coronal view depicting a midline large extracalvarial metastatic lesion over the Bregma.

**FIGURE 2:**
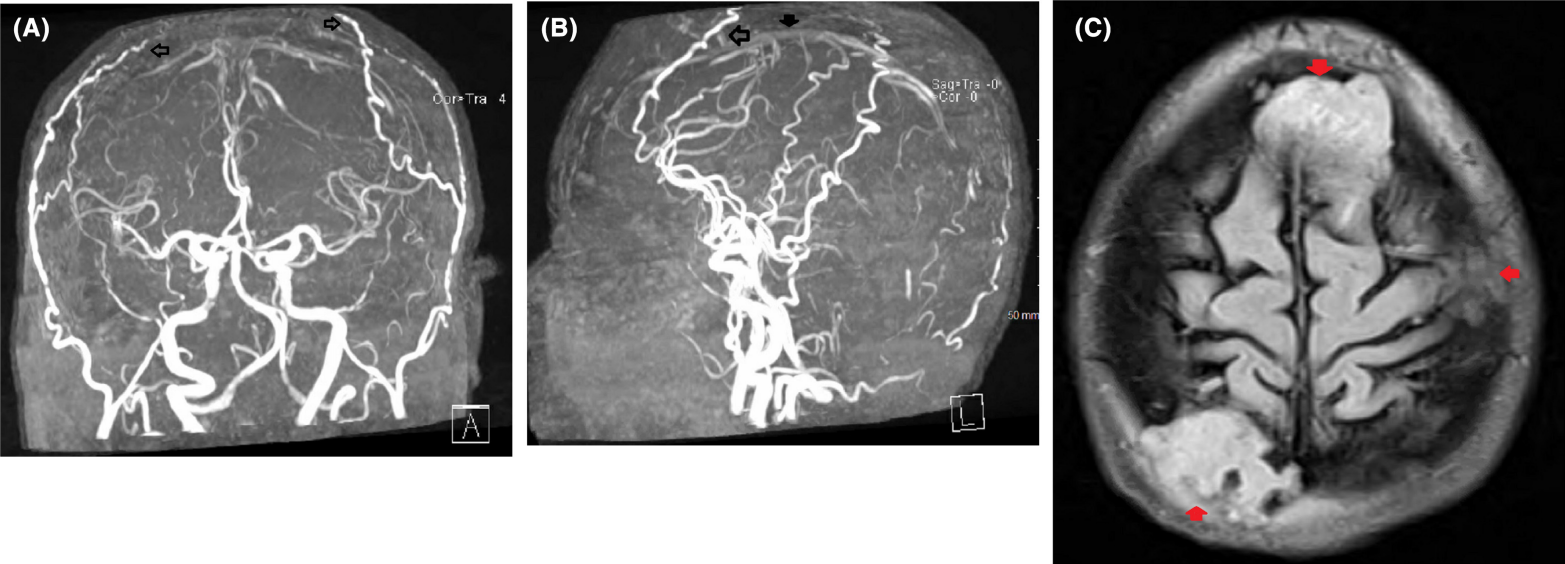
(A) Preoperative MRV (arterial phase) coronal sequence demonstrating the feeders from bilateral superficial temporal artery. (B) Preoperative MRA (arterial phase) sagittal sequence demonstrating the feeders from bilateral superficial temporal artery. The sagittal sinus appears to be compressed (filled arrow). (C) Preoperative MRI brain with and without contrast axial T2 sequence revealing extra‐axial lesions (bregma, larger right parietal, and the smaller left parietal).

Preoperative head computed tomography (CT) scan without contrast revealed multiple calvarial lytic lesions extending between the extra‐axial space overlying the bregma and high on cerebral convexity predominantly and the overlying the soft tissues (Figure 1A,B). Magnetic resonance venography (MRV) revealed compressed but patent superior sagittal sinus that was not thrombosed (Figure 2A,B). Magnetic resonance imaging (MRI) also revealed that the largest lesion was in the bifrontal region around the bregma (Figure 2C), suggesting a substantial hemorrhagic component within this sizable lesion.

### 2.3 Surgical technique

We performed successful neurointerventional embolization of external carotid artery (ECA) branch superficial temporal artery (bilateral frontal and parietal branches) supplying the bifrontal and biparietal metastatic calvarial and extra‐calvarial lesions two days prior to the planned surgery. Preoperative medical optimization included hemodialysis. The patient was positioned supine, and we established a right‐sided arterial line and left external jugular IV access. We employed neuronavigation using Medtronic Stealth to confirm the lesion's location against the preoperative MRI scan.

**FIGURE 3:**
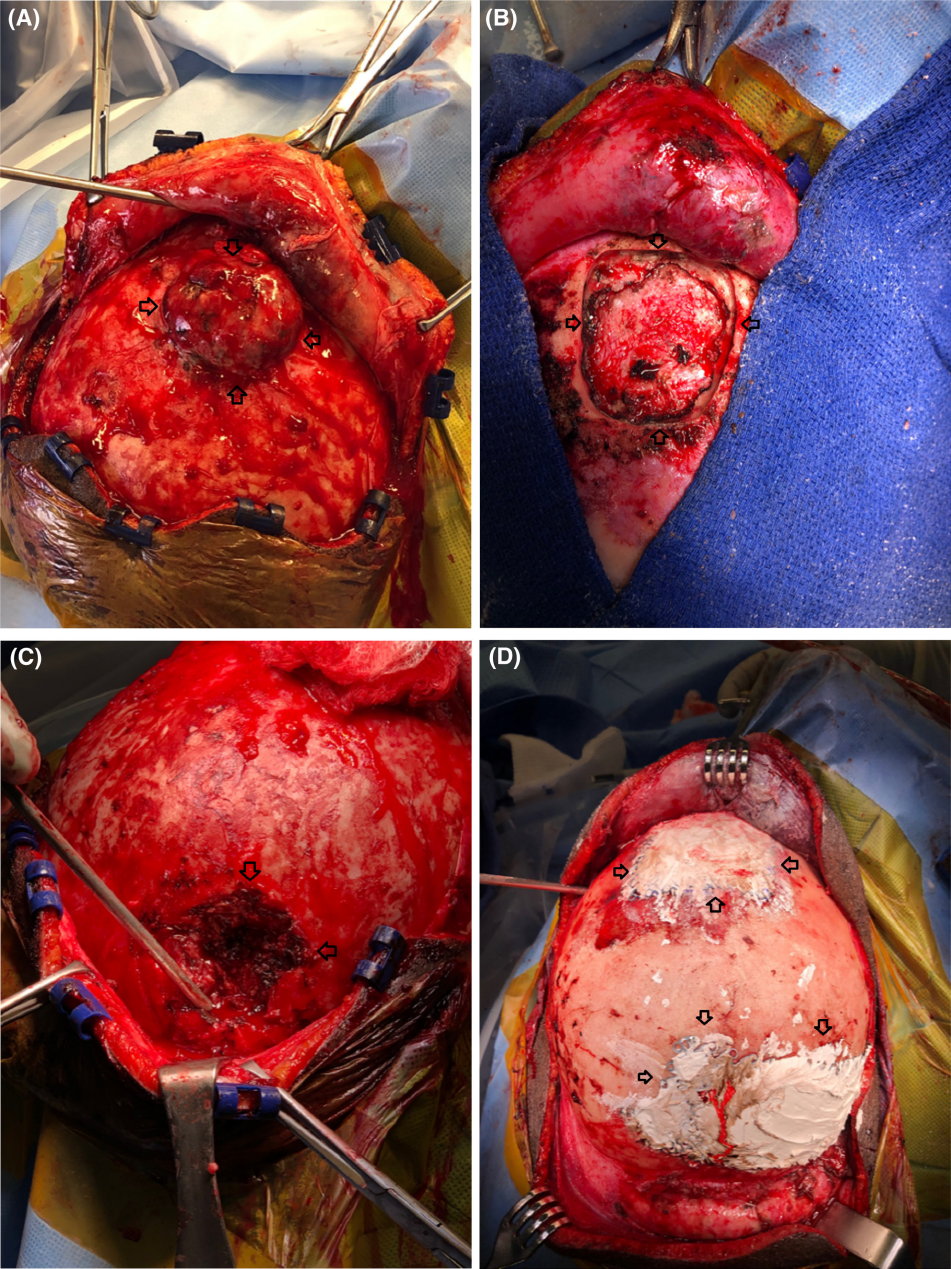
(A) Intraoperative image revealing the large fungating hemorrhagic metastatic lesion around the bregma after the scalp margin was reflected anteriorly. (B) Intraoperative image after the anterior lesion around the bregma with gross total resection. (C) Right‐sided larger parietal hemorrhagic lesion is being resected out. (D) After resection of all lesions cranioplasty was performed with the use of Titanium mesh with the polymethymethacrylate bone cement.

Bi‐coronal incision around the calvarial metastases revealed a round metastatic tumor measuring 5 × 5 cm, located intracalvarial with significant extracalvarial component, was identified around bregma (Figure 3A). Utilizing bipolar cautery, we performed a circumferential blunt dissection technique around the anterior frontal tumor. The tumor was highly hemorrhagic, requiring careful coagulation of superficial capsular vessels. We devitalized the extracalvarial metastatic tumor circumferentially without any injury to the sagittal sinus. The tumor, adherent to the periosteal dura, was dissected out completely using an ultrasonic aspirator (Sonopet). Bony margins were smoothed with a multi‐fluted cutting burr, and attention was given to larger right‐sided parietal metastasis using a similar technique. (Figure 3B,C).

In the left parietal metastasis, we identified a minimal amount of soft tissue tumor. Hemostasis was completely secured, and the calvarial invasion appeared to be completely resected and drilled. We used Sonopet was also to resect any residual metastatic tumor; however, we used bipolar electrocautery to resect the whole tumor. We traversed all bone‐tumor interface, and hemostasis around the bony margins, as well as the tumor base, was secured with the use of thrombin‐soaked gel‐foam as well as the Floseal. Appropriately sized, we cut two pieces of titanium mesh to cover the three bony defects. We used 5 mm and 4 mm screws to secure the titanium mesh to the calvarium. Later, we applied polymethymethacrylate (HydroSet) bone cement to cover the titanium mesh over the bony defects (Figure 3D). The patient was brought to the ICU for further observation.

### 2.4 Postoperative course

The patient endorsed continued improvement and recovery from her surgery. Postoperative head CT scan without contrast has revealed gross total resection of the extra‐ and calvarial lesions with clean dural base as well margins of the resection cavity. The patient recovered quickly and was discharged in a stable condition on postoperative day (POD) 5.

Histopathological diagnosis from intraoperative biopsy confirmed high‐grade clear cell carcinoma, which focally invaded the bone. Prominent areas of necrosis were present. No sarcomatous or rhabdoid features were identified. The tumor cells stain positive for PAX8, CD10, and vimentin.

At 4‐week postoperative follow‐up, the patient documented normal neurological examination. Scalp wound was healing well except 1 × 1 cm area with a pale border and some darkening on the right temporal area likely due to poor scalp perfusion with use of pre‐operative bilateral superficial temporal artery embolization. Plastic surgery was therefore immediately consulted, and close monitoring was therefore recommended.

**FIGURE 4:**
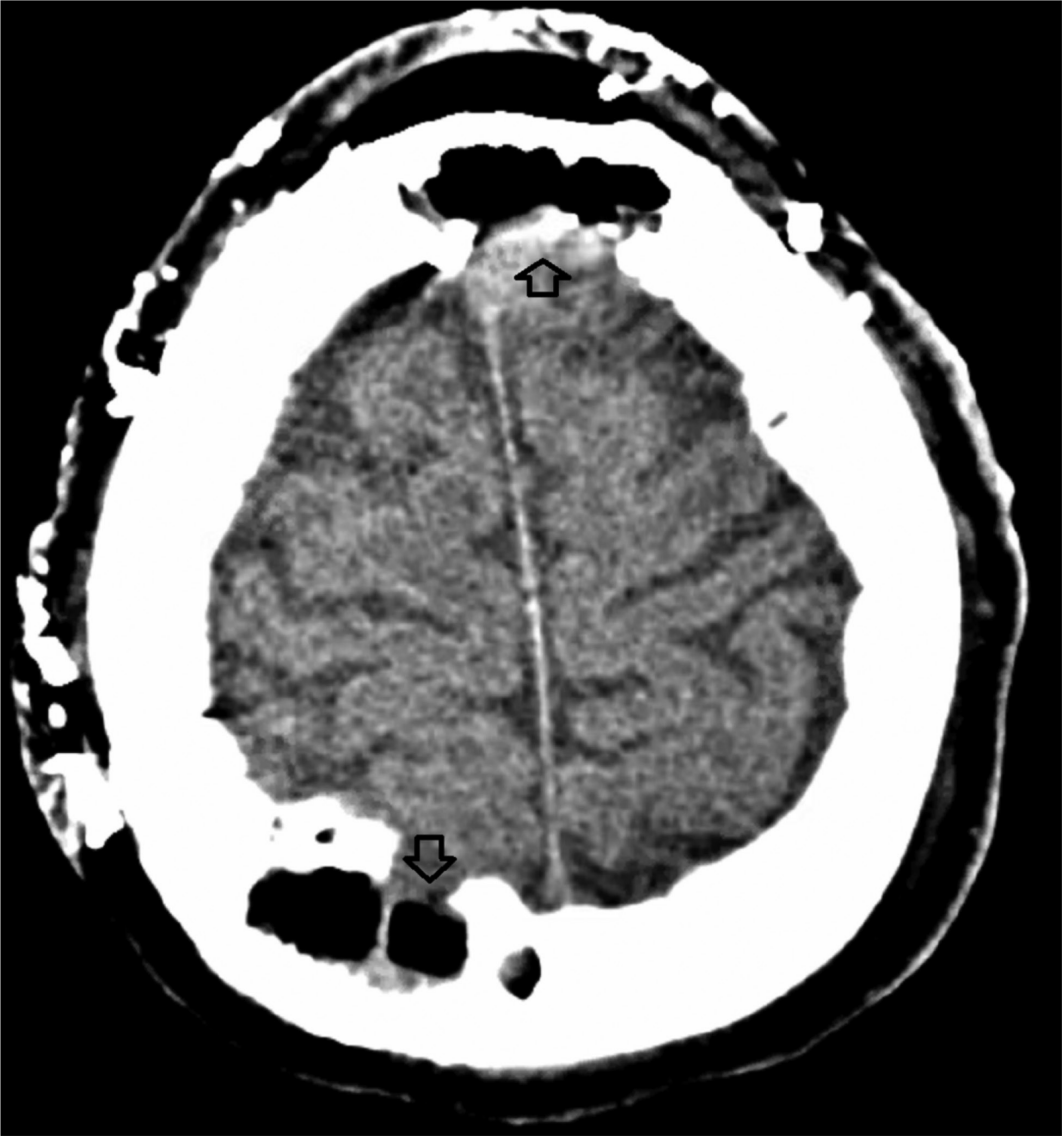
Head CT scan without contrast axial view at 3‐month postoperative follow‐up visit revealing a gross total resection of all calvarial lesions.

**FIGURE 5:**
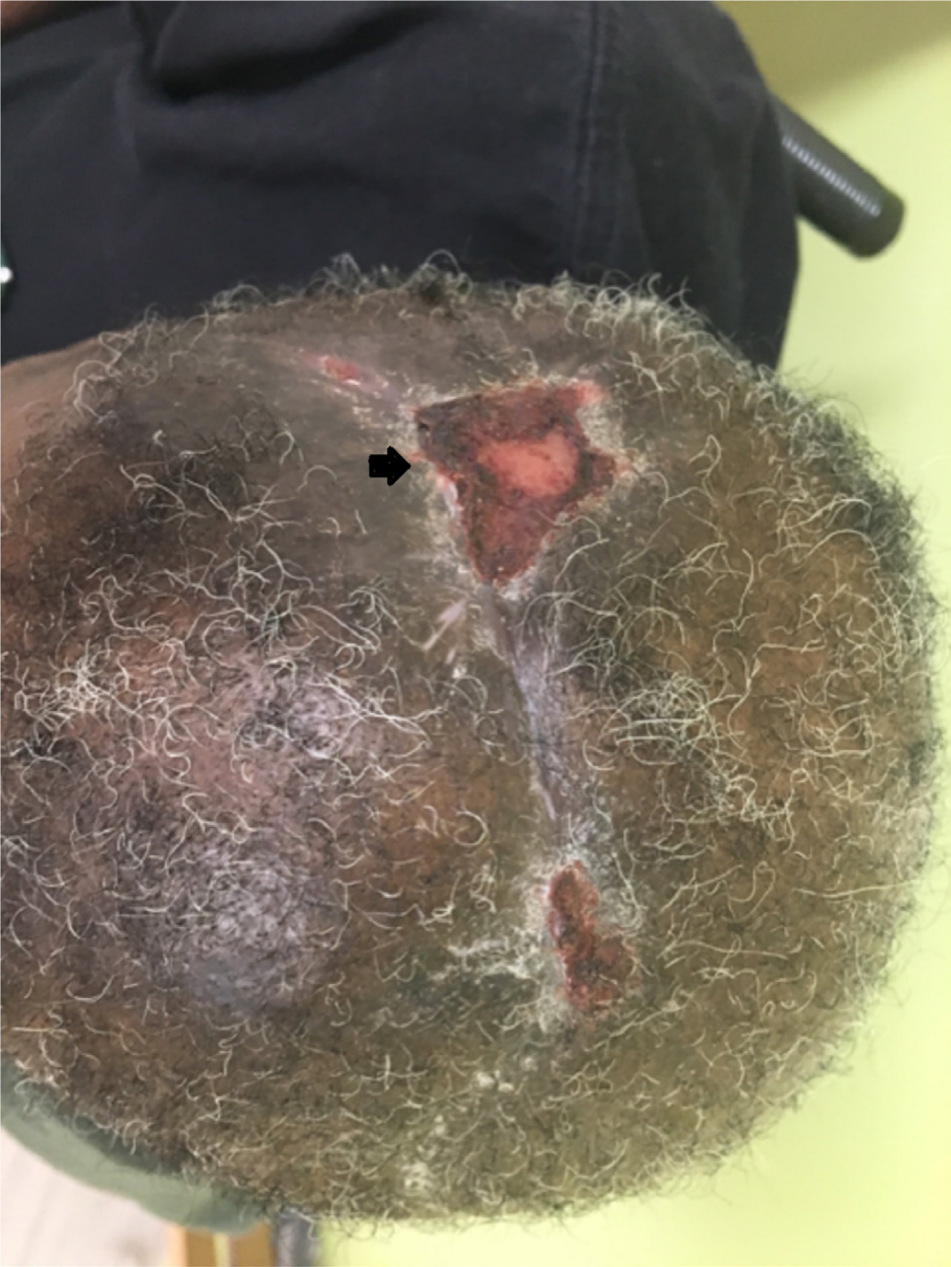
Photograph image at 3‐month postoperative follow up with an area of delayed healing on the right aspect of the wound margin.

At 3‐month follow‐up, head CT scan without contrast confirmed no interval recurrence (Figure 4), no signs of wound necrosis, or an infection were observed (Figure 5).

**FIGURE 6:**
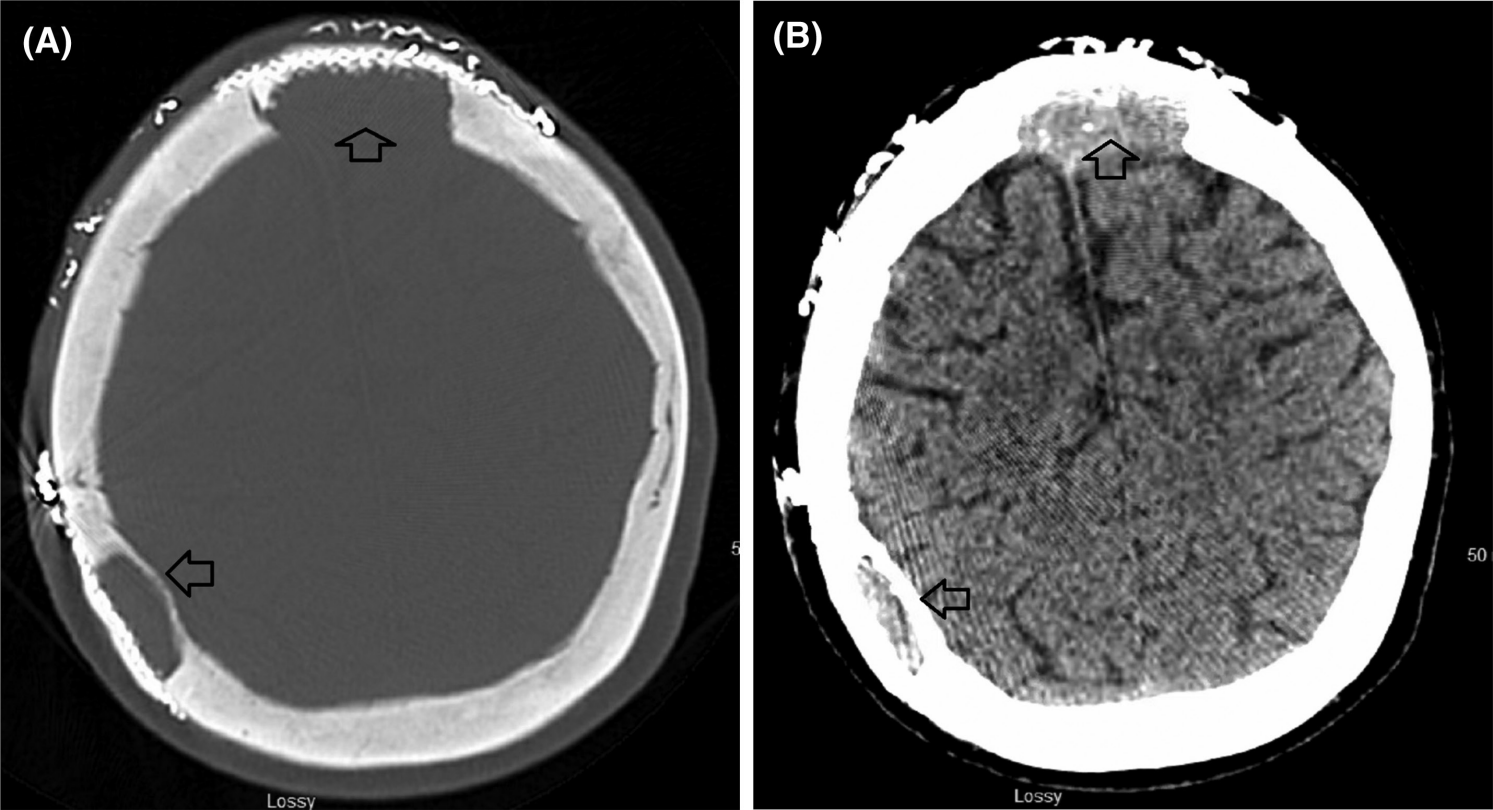
(A) Head CT scan without contrast axial view (bone‐window) at 6‐month postoperative follow‐up visit revealing an intact Titanium mesh implants around the site of bony resections. (B) Head CT scan without contrast axial view at 6‐month postoperative follow‐up visit revealing a gross total resection of all calvarial, and extracalvarial lesions.

**FIGURE 7:**
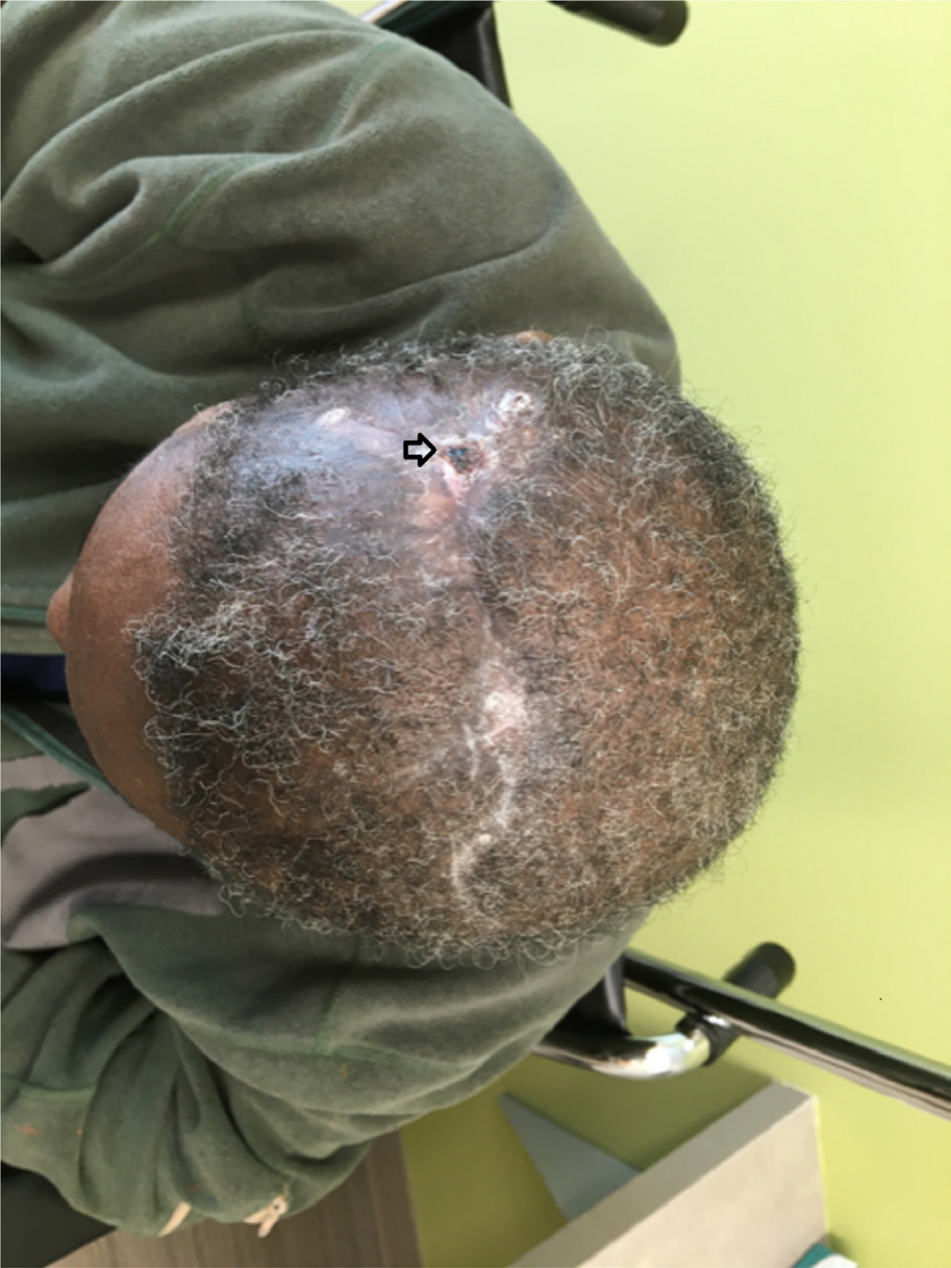
Photograph image at 6‐month follow‐up revealing complete healing of the scalp wound with the minimal scabbing.

At 6‐month follow‐up visit, head CT scan without contrast remained stable without any recurrence (Figure 6A,B). The scalp incision appeared to have healed well with minimal scabbing around the delayed healing site area (Figure 7).

## 3 DISCUSSION

Renal cell carcinoma is among 10 most common worldwide cancers and can have devastating outcomes. Of the three major cancer cell subtypes, clear cell renal cell carcinoma is the most common and has the highest metastatic potential. Unfortunately, CCRCC can still have poor prognosis following nephrectomy due to about 30% of patients still experiencing metastatic disease.^2^, ^4^, ^16^, ^17^, ^18^ Although the systemic disease burden at an initial presentation is unpredictable due to incidental identification and the diagnosis.^1^ The management of renal cell metastases is still an ongoing concern. Therefore, a definitive approach to treatment depends on the patient's overall medical condition, systemic disease control, and the extent of metastatic disease.

Advanced disease may require systemic drug therapy such as tyrosine kinase inhibitors.^17^ Before systemic treatment, metastatic brain lesions can be addressed locally with the surgical resection, stereotactic radiosurgery, or with whole‐brain radiation therapy.^19^ In the setting of symptomatic scalp lesions with swelling, metastasectomy may be locally curative for the metastases and is generally recommended.^4^ Approach to metastasectomy varies based on site/location of these lesions. Three most common sites of metastasis are lung, bone, brain, and the lymph nodes. Calvarium is one of the rarest sites of metastasis. The spinal epidural venous plexus (Batson's Plexus) is an important route of metastasis to the head and neck along with the general hematogenous route.^18^ Metastases that travel through Batson's Plexus may explain the calvarial lesions.

Calvarial metastases typically originate from cancers of the lungs, breasts, prostate, or thyroid. Metastasis to the calvarium from primary renal carcinoma is unusual and generally is not a typical systemic presentation.^11^, ^12^, ^13^, ^14^, ^15^ Symptomatic skull masses as reported in our case are indicative of metastatic calvarial lesions. Over the past 30 years, the literature has documented limited instances of renal cell carcinoma (RCC) presenting with calvarial metastases.^5^, ^10^, ^11^, ^12^, ^13^, ^14^, ^15^, ^16^, ^18^, ^20^, ^21^, ^22^, ^23^ (refer to Table 1).

**TABLE 1:** Literature review on metastatic renal cell carcinoma with a focus on the initial manifestation involving the calvaria.

Study ID	Patient no.	Sex/age	Presentation	Localization	Oncologic treatment	Surgical treatment	Outcomes
Zhang WQ 2018^15^	1	M/54	Palsies of right cranial nerves III, IV, VI, and V2	Clivus and bilateral petrous apexes	Gamma knife	None	Not‐improved
Pang 2018^23^	1	M/41	Calvarial haemangioma	Left frontotemporal	High dose IL‐2	Yes	Improved—No recurrence or residual
Badri 2018^22^	1	M/65	Skull mass	Left parietal	Adjuvant chemotherapy	Yes	Improved—No recurrence or residual
Suffee 2016^14^	1	M/62	Skull mass	Right parietotemporal	Radiation, chemotherapy and antiangiogenetic therapy	Yes	After palliative care: Not improved After Surgery: improved, no recurrence, satisfactory cosmetic outcome
Ouma 2017^16^	1	F/11	Palsy of left sixth nerve	Left parietal	Palliative care	None	Not Improved Death in 1 month
Mani A 2017^20^	1	M/55	Holocranial headache and diplopia	Left parietal	External beam radiation therapy (EBRT)	None	Not Improved Death in 6 weeks
Gaur K 2015^10^	1	F/17	Headache and Skull mass	Frontoparietal	None	None	Not‐Improved Death
Jindal T 2014^11^	1	F/37	Skull masses	Bilateral frontal	None	None	Not‐Improved Death in 3 weeks
Rekhi B 2009^12^	1	F/15	Skull mass	Occipital	Palliative chemotherapy	None	Not Improved Death in 5 months
Yeh HC 2008^7^	1	M/80	Skull mass	Parietal	Chemotherapy	Yes	Improved (22 months follow‐up)
De Vos C 2005^9^	1	–	Skull mass	Temporal	Radiotherapy	Yes	Improved
Gaetani P 2004^13^	2	M/66 F/60	Skull mass Skull mass	Occipital Frontal	Yes Yes	Yes Yes	Improved (7 months follow up for both)
Koutnouya 1998^21^	1	F/33	Skull mass	Frontal	No	No	Death in 9 months
Wahner 1997^18^	1	F/72	Skull mass	Occipital	Radiotherapy	No	Improved (15 months of follow‐up)

Notably, in 12 of the cases including our case, the calvarial metastasis was the first indication of RCC involvement. (Table 1) Other cases have presented with additional manifestations, such as cranial nerves palsies^15^, ^16^, ^20^ Furthermore, the metastatic disease has the potential to affect all calvarial bones and extend beyond the calvarium. Bilateral cases were also observed.^11^, ^15^ The majority of patients affected were elderly people, with some notable instances of involvement in younger individuals.^10^, ^12^, ^16^

Calvarial metastases in patients are typically associated with advanced stages of disease, and diagnosis can be challenging due to often subtle symptoms. In fact, these metastases are commonly discovered during autopsies,^11^, ^13^ and only a limited number of studies have delved into their clinical presentation. Treatment of calvarial metastasis can be challenging and complex. Aside from diagnostic, curative, and preventive surgeries, surgical procedures are also undertaken to enhance the quality of life in these patients.^14^ Furthermore, there is no perioperative morbidity, as demonstrated in our case similar to previously reported case studies. Complete resection may also reduce recurrence if, compared to palliative or oncologic care alone. Since a significant portion of these lesions is associated with scalp pain, cosmetic issues, recurrent events leading to bleeding and infection. Calvarial and extracalvarial disease can significantly compromise quality of life while greatly impacting social life, a complete resection is therefore recommended.

The best approaches to palliative treatment are still being worked out. En bloc resection has been shown to be effective in treatment of calvarial or extracalvarial metastases despite dural sinus involvement.^24^ Gamma Knife surgery is also documented to be effective in treating a selected group of patients with the calvarial metastases^11^, ^13^, ^14^; although there may be risk of cerebrospinal fluid (CSF) leakage with the invasive calvarial lesions. Our approach included a bicoronal incision with the bifrontal as well as biparietal craniectomies for the resection of the patient's extra‐ and calvarial metastasis. There were no complications observed during or after the surgery, but only the delayed healing of a small area of scalp, away from the incision that occurred due to preoperative aggressive use of bilateral superficial temporal artery embolization. Maybe this complication can be avoided by an isolated unilateral major arterial feeder embolization. Conversely, this preoperative additive technique has been helpful to minimize the significant intraoperative blood loss, which could have been encountered during surgery. Authors support that although bilateral embolization is associated with a risk, however, there is not an effective method to combat the inadvertent blood loss considering the patency of the arteriovenous anastomosis embedded within the tumor. While instituting preoperative hemostatic control, we believe our surgical technique was effective in treating renal cell carcinoma calvarial metastases and has the potential to treat similar metastases of other primary cancer types.

## 4 CONCLUSIONS

Extra‐ and the calvarial metastasis from systemic venous spread of renal cell tumors is an extremely rare presentation. The treatment of these metastatic lesions can be very challenging. Considering, an inherent radioresistant nature of the renal cell tumor, a palliative surgical resection can be offered to control metastatic spread and improve the quality of life of these patients. Thus, surgical planning is crucial in these cases. Selective preoperative embolization of arterial feeders can be utilized to help decrease intraoperative blood loss but can pose risk of poor wound healing. Careful and controlled resection around sagittal sinus is strongly recommended to avoid sinus injury. After resection, cranioplasty with titanium mesh and bone cement is a viable treatment option, moreover, could help relieve symptomatic agonizing scalp pain, offers better cosmesis, and improves overall quality of life. In summary, palliative surgical resection of extra‐ and/or calvarial metastatic lesions helps to control metastatic disease and prevents associated morbidity if these lesions are left untreated.

## AUTHOR CONTRIBUTIONS

**Rahim Abo Kasem:** Conceptualization; data curation; formal analysis; investigation; methodology; resources; validation; visualization; writing – original draft; writing – review and editing. **Karan Joseph:** Conceptualization; data curation; formal analysis; investigation; methodology; validation; visualization; writing – original draft; writing – review and editing. **Adnan Shaik:** Conceptualization; data curation; formal analysis; investigation; methodology; validation; visualization; writing – original draft; writing – review and editing. **Angela Downes:** Investigation; methodology; validation; writing – review and editing. **M. Burhan Janjua:** Conceptualization; data curation; formal analysis; investigation; methodology; project administration; resources; supervision; validation; visualization; writing – original draft; writing – review and editing.

## FUNDING INFORMATION

There are no funding sources to report for this case report.

## CONSENT

Written informed consent was obtained from the patient to publish this report in accordance with the journal's patient consent policy.

## Data Availability

We do not have any research data outside the submitted manuscript file.
